# Outcomes of Nucleos(t)ide Analogue Discontinuation in Noncirrhotic Patients With Chronic Hepatitis B: A Retrospective Study

**DOI:** 10.7759/cureus.106047

**Published:** 2026-03-29

**Authors:** Shrey Bhatt, Ujjwal Sonika, Venkatesh Vaithiyam, Siddharth Srivastava, Deep Patel, Ashok Dalal, Ajay Kumar, Barjesh Chander Sharma, Sanjeev Sachdeva

**Affiliations:** 1 Gastroenterology, Govind Ballabh Pant Institute of Postgraduate Medical Education and Research, New Delhi, IND

**Keywords:** apasl, chronic hepatitis b infection, nucleos(t)ide analogue cessation, sustained remission, virological relapse

## Abstract

Background

Finite nucleos(t)ide analogue (NUC) therapy is an emerging treatment strategy in selected patients with chronic hepatitis B (CHB). We evaluated the feasibility, efficacy, and safety of NUC discontinuation in non-cirrhotic patients fulfilling the Asian Pacific Association for the Study of the Liver (APASL) stopping criteria.

Methods

We retrospectively analysed data from a tertiary gastroenterology clinic (January 2021-December 2024). Consecutive non-cirrhotic CHB patients meeting APASL criteria were included: hepatitis B e-antigen (HBeAg)-positive with stable HBeAg seroconversion and undetectable hepatitis B virus (HBV) DNA ≥12 months (preferably ≥3 years), or HBeAg-negative with ≥2 years of therapy and undetectable HBV DNA on three occasions, six months apart. Exclusion criteria were significant alcohol intake, viral coinfections, extrahepatic HBV disease, personal or family hepatocellular carcinoma (HCC) history, cirrhosis by imaging or elastography, or unwillingness to discontinue therapy. Patients were monitored for two years with clinical assessment, alanine aminotransferase (ALT), and HBV DNA. Retreatment was predefined upon virological or biochemical relapse.

Results

Twenty-nine patients were included (HBeAg-positive, 15; HBeAg-negative, 14). At three months, virological relapse occurred in 14/29 (48.3%) patients, more frequently in HBeAg-positive than in HBeAg-negative patients (10/15 (66.7%) vs 4/14 (28.6%); p = 0.047). No significant difference in virological relapse was observed between patients receiving tenofovir and those receiving entecavir (ETV) (5/15 (33.28%) vs 9/14 (64.28%); p = 0.09). All relapsed patients restarted the same NUC and achieved complete viral suppression within two years. Sustained off-therapy remission was observed in 15/29 (51.7%), more common in HBeAg-negative than HBeAg-positive patients (10/14 (71.4%) vs 5/15 (33.3%); p = 0.049). No flare, hepatic decompensation, antiviral non-response, or deaths occurred.

Conclusions

Finite NUC therapy was feasible and safe in non-cirrhotic CHB patients meeting APASL criteria. Sustained remission was maintained in half of the patients, supporting finite therapy with early monitoring and clear retreatment thresholds. However, larger multicentre studies are needed to validate these findings.

## Introduction

Chronic hepatitis B virus (HBV) infection is a leading global cause of chronic liver disease, cirrhosis, and hepatocellular carcinoma (HCC) [1]. Potent nucleos(t)ide analogue (NUC) agents, including entecavir (ETV) and tenofovir disoproxil fumarate/tenofovir alafenamide (TDF/TAF), achieve sustained viral suppression, reduce hepatic necroinflammation, and slow progression to advanced liver disease [2,3]. Despite these benefits, functional cure, defined as the loss of hepatitis B surface antigen (HBsAg), is rarely achieved during ongoing therapy, requiring prolonged or indefinite treatment in most cases [4,5].

The cumulative treatment burden, risks of long-term toxicities, and adherence challenges have increased interest in finite therapy strategies for selected patients [6]. The Asian Pacific Association for the Study of the Liver (APASL) recommends discontinuation of NUCs in non-cirrhotic, hepatitis B e-antigen (HBeAg)-negative patients after ≥2 years of therapy, provided that HBV DNA remains undetectable on three separate occasions at least six months apart [6]. Recent multicentre trials, cohort studies, and systematic reviews have assessed virological and biochemical relapse rates, HBsAg loss, and safety profiles following treatment cessation [1-5,7].

Beyond APASL, major Western societies differ in the extent to which they endorse finite NUC therapy. The AASLD (2018) is more conservative, generally favouring continued therapy in HBeAg-negative patients unless there is a compelling rationale and robust post-cessation monitoring capacity [8]. EASL guidance (2017) likewise emphasised individualised decisions with close surveillance, and its 2025 update refines monitoring schedules, reiterating that selected non-cirrhotics may be considered for cessation when stringent criteria are met and retreatment thresholds are predefined [9,10].

Epidemiologically, India contributes a substantial share of the global HBV burden, with general-population HBsAg prevalence commonly reported around 1.5%-3%, but varying by region and sub-population [11,12]. Recent Indian work also underscores programmatic progress (e.g., improved birth-dose coverage and declining paediatric HBsAg prevalence), yet highlights persistent heterogeneity across states and tribal communities [13]. These contextual data support the relevance and feasibility of structured finite-therapy strategies within Indian hepatology practice, provided that systems for rapid relapse detection and retreatment are in place.

This study investigated the clinical and virological outcomes of NUC withdrawal in non-cirrhotic patients with chronic hepatitis B (CHB) who met the APASL criteria, focusing on early relapse kinetics, retreatment patterns, and safety, and placing these findings in the context of existing evidence.

## Materials and methods

**Figure 1:**
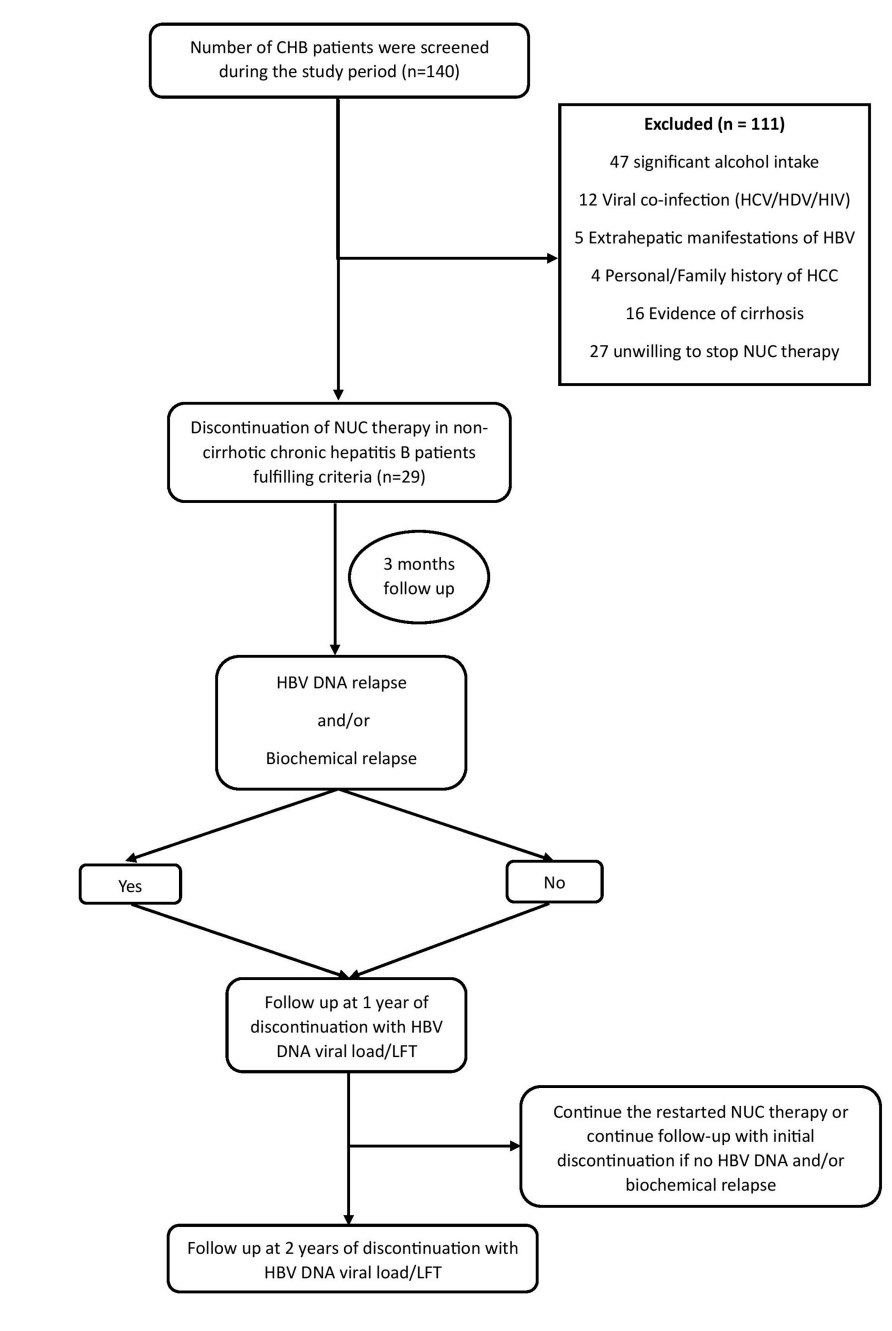
Study Design and Follow-Up Algorithm for NUC Discontinuation in Non-cirrhotic Chronic Hepatitis B CHB: Chronic Hepatitis B; NUC: Nucleos(t)ide Analogue; HCV: Hepatitis C Virus; HDV: Hepatitis D Virus; HIV: Human Immunodeficiency Virus; HBV: Hepatitis B Virus; HCC: Hepatocellular Carcinoma; LFT: Liver Function Test

This retrospective data analysis was conducted in the Department of Gastroenterology at Govind Ballabh Pant Institute of Post-Graduate Medical Education and Research, New Delhi, India, from January 2021 to December 2024. Clinical, laboratory, and treatment-related data were extracted from the medical records of patients attending the liver clinic at our institution and reviewed for analysis. A total of 29 consecutive, non-cirrhotic CHB patients who fulfilled the APASL-recommended criteria for NUC discontinuation were included (Figure 1).

Patients were selected for NUC discontinuation based on APASL-recommended criteria [6]. HBeAg-positive patients were eligible for therapy stoppage if they had achieved stable HBeAg seroconversion, with undetectable HBV DNA for at least 12 months - preferably for ≥3 years - prior to discontinuation. HBeAg-negative patients were considered eligible for therapy discontinuation after receiving at least two years of NUC therapy, with undetectable HBV DNA documented on three separate occasions, each six months apart. Patients were excluded if they had a history of significant alcohol intake, co-infection with hepatitis C virus (HCV), hepatitis D virus (HDV), or human immunodeficiency virus (HIV), a history of extrahepatic manifestations of hepatitis B, or a personal or family history of HCC. Additional exclusion criteria included ultrasound evidence of chronic liver disease, a liver stiffness measurement (FibroScan) greater than 12.5 kPa, and unwillingness to discontinue NUC therapy.

All patients had structured monthly follow-up visits for clinical evaluation during the first three months, then every three months until one year, and every six months up to two years after discontinuation. HBV DNA and liver function tests were done at 3, 6, 12, and 24 months after discontinuing the medication. In cases of virological and/or biochemical relapse, as per APASL guidelines, previously used NUC therapy was restarted. Virological relapse is defined as a rise in HBV DNA to ≥2,000 IU/mL after stopping antiviral therapy. A biochemical (clinical) relapse is characterised by HBV DNA ≥2,000 IU/mL, accompanied by an increase in alanine aminotransferase (ALT) to more than twice the upper limit of normal (ALT > 2× ULN) [7].

Statistical analysis

Data were analysed using IBM SPSS Statistics for Windows, Version 26 (Released 2018; IBM Corp., Armonk, NY, USA). Continuous variables are presented as mean ± standard deviation (SD) or median (lower and upper quartiles), and categorical variables as frequencies and percentages. Group comparisons between HBeAg-positive and negative patients were performed using the Mann-Whitney U test for continuous variables and Fisher’s exact test for categorical variables. A p-value <0.05 was considered statistically significant.

## Results

A total of 29 non-cirrhotic CHB patients (18 males) who met APASL discontinuation criteria were included. Of these, 15 (51.7%) were HBeAg-positive and 14 (48.3%) were HBeAg-negative at baseline (Table 1). Fourteen (48.3%) patients required retreatment. The baseline comparison and liver function tests at the time of treatment stoppage, between those who required retreatment and those who did not, are shown in Table 1.

**Table 1:** Baseline Characteristics Continuous variables are presented as median and IQR, while categorical variables are shown as n (%). HBeAg: Hepatitis B e-Antigen; AST: Aspartate Aminotransferase; ALT: Alanine Aminotransferase; HBV DNA: Hepatitis B Virus DNA

	Total (N = 29)	Retreatment started (n = 14)	Retreatment not started (n = 15)
Demographics			
Males	18 (62%)	8 (57.14%)	10 (75%)
Females	11 (38%)	6 (42.8%)	5 (25%)
Median Age (Years)	24 (16-33)	24 (16-28)	25 (16-44)
Baseline Liver Function Tests (LFT)			
Median Serum Bilirubin (mg/dL)	0.7 (0.5-1)	0.6 (0.3-0.7)	0.8 (0.6-1.3)
Median AST (U/L)	69 (36.5-108.5)	45.5 (33-92)	110 (58-346)
Median ALT (U/L)	83 (47.5-151.5)	57 (35-83)	126 (79-400)
Total Protein (gm/dL)	7.4 (7-7.8)	7.3 (7-7.7)	7.45 (7-7.8)
Albumin (gm/dL)	4.1 (3.81-4.3)	4.1 (3.7-4.3)	4.15 (3.9-4.3)
Baseline Hepatitis B Serology			
HBeAg Positive Patients at Baseline	15 (51.72%)	10 (71.4%)	5 (33.3%)
HBeAg Negative Patients at Baseline	14 (48.28%)	4 (28.57%)	10 (66.67%)
Median HBV DNA (IU/mL)	115000 (22750-7300000)	764500 (18000-10000000)	112000 (27500-4600000)
Liver Function Test at the Stoppage of Treatment			
Median S. Bilirubin (mg/dL)	0.5 (0.3-1)	0.5 (0.4-0.6)	0.5 (0.4-0.9)
Median AST (U/L)	25 (14-38)	24.5 (20-26)	25 (23-30)
Median ALT (U/L)	27 (13-42)	26.5 (18-32)	32 (24-34)
Total Protein (gm/dL)	7.5 (7.1-7.7)	7.5 (7.1-7.6)	7.5 (7.1-7.9)
Albumin (gm/dL)	4.4 (4.1-4.7)	4.4 (4.2-4.7)	4.5 (4.1-4.7)

Three months after stopping treatment, HBV DNA reactivation was observed in 14 patients (48.28%). Reactivation was more common in HBeAg-positive patients (10/15; 66.7%) than in HBeAg-negative patients (4/14; 28.6%), with a statistically significant difference (p = 0.047, Fisher’s exact test). None of the patients experienced a biochemical flare. The HBV DNA status at various time points after treatment stoppage in HBeAg-positive patients is shown in Table 2. At 24 months, all patients who required retreatment again became DNA-negative (Table 2).

**Table 2:** HBV DNA Status After Nucleos(t)ide Analogue Discontinuation Among HBeAg-Positive Patients *Patients with HBV DNA reactivation at three months post-NUC discontinuation were re-initiated on the same antiviral therapy. NUC: Nucleos(t)ide Analogue; HBeAg: Hepatitis B e-Antigen; HBV DNA: Hepatitis B Virus DNA

HBV DNA status	Negative	Positive
3 months (n (%))	5 (33.33)	10 (66.67)*
12 months (n (%))	12 (80)	3 (20) (no biochemical flare)
24 months (n (%))	15 (100)	0

The HBV DNA status at various time points after treatment stoppage in HBeAg-negative patients is shown in Table 3. At 24 months, all patients who required retreatment again became DNA-negative (Table 3).

**Table 3:** HBV DNA Status After NUC Discontinuation Among HBeAg-Negative Patients *Patients with HBV DNA reactivation at three months post-NUC discontinuation were re-initiated on the same antiviral therapy. NUC: Nucleos(t)ide Analogue; HBeAg: Hepatitis B e-Antigen; HBV DNA: Hepatitis B Virus DNA

HBV DNA status	Negative	Positive
3 months (n(%))	10 (71.42)	4 (28.58)*
12 months (n(%))	13 (92.85)	1 (7.14) (no biochemical flare)
24 months (n(%))	14 (100)	0

All patients who experienced reactivation had their previous NUC regimen reinstated. All re-treated patients achieved HBV DNA suppression within the two-year follow-up period, without experiencing biochemical flares or hepatic decompensation. The follow-up DNA levels at various time points among HBeAg-positive and HBeAg-negative patients are shown in Tables 4-5, respectively.

**Table 4:** Follow-Up HBV DNA Levels in HBeAg-Positive Patients HBeAg: Hepatitis B e-Antigen; HBV DNA: Hepatitis B Virus DNA

Follow-up	DNA (IU/mL) (median (range))
3 months (n = 10)	55000000 (1620-100000000)
6 months (n = 3)	63400 (27000-150000)
12 months (n = 3)	765 (700-1300)

**Table 5:** Follow-Up HBV DNA Levels in HBeAg-Negative Patients HBeAg: Hepatitis B e-Antigen; HBV DNA: Hepatitis B Virus DNA

Follow-up	DNA (IU/mL) (median (range))
3 months (n = 4)	26195 (3125-1400000)
6 months (n = 3)	3500 (880-6800)
12 months (n = 2)	300 (250-350)

Among baseline HBeAg-positive patients, the median durations of HBeAg negativity, HBV DNA negativity, and total NUC therapy did not differ significantly between those who experienced HBV DNA reactivation and those who maintained HBV DNA negativity at three months after NUC discontinuation (Table 6).

**Table 6:** Comparison of HBeAg Status, Duration of HBV DNA Negativity, and Treatment Between Patients With and Without HBV DNA Positivity Among HBeAg-Positive Patients at 3 Months of Follow-Up Comparisons between groups were performed using the Mann-Whitney U test. HBeAg: Hepatitis B e-Antigen; HBV DNA: Hepatitis B Virus DNA; NUC: Nucleos(t)ide Analogue

HBV DNA status (after 3 months of discontinuation of NUC therapy)	Negative (n = 5)	Positive (n = 10)	Test statistic	p-value
HBeAg negativity duration before stopping NUC (median; IQR in months)	20 (12-47)	46 (21-84)	11	0.83
HBV DNA negativity duration before stopping NUC (median; IQR in months)	20 (15-46)	36 (18-60)	18	0.461
Total duration of NUC therapy (median; IQR in months)	38 (34-77)	88 (45-120)	12	0.95

Similarly, among baseline HBeAg-negative patients, the median duration of HBV DNA negativity and the total duration of NUC therapy were comparable between patients with HBV DNA reactivation and those who remained HBV DNA-negative at three months post-discontinuation (Table 7).

**Table 7:** Comparison of the Duration of HBV DNA Negativity and Treatment Between Patients With and Without HBV DNA Positivity, Among HBeAg-Negative Patients Comparisons between groups were performed using the Mann-Whitney U test. HBeAg: Hepatitis B e-Antigen; HBV DNA: Hepatitis B Virus DNA; NUC: Nucleos(t)ide Analogue

HBV DNA status (after 3 months of discontinuation of NUC therapy)	Negative (n = 10)	Positive (n = 4)	Test statistic	p-value
HBV DNA negativity duration before stopping NUC (median; IQR in months)	35 (18-45)	35.5 (25-48)	19	0.887
Total duration of NUC therapy (median; IQR in months)	52 (42-62)	60 (49.5-60)	17	0.667

Fifteen patients (51.72%) remained in sustained virological remission throughout the two-year follow-up period. Sustained remission was more common in HBeAg-negative patients (10/14; 71.4%) than in HBeAg-positive patients (5/15; 33.3%), with this difference reaching statistical significance (p = 0.049, Fisher’s exact test).

When analysed by drug type, relapse requiring retreatment was more frequent among patients who had discontinued ETV compared with tenofovir. Specifically, 9/14 (64.3%) patients previously on ETV required retreatment, compared to 5/15 (33.3%) patients previously on Tenofovir (p = 0.09). Although these differences did not reach conventional statistical significance, they suggest that both HBeAg-positive status and prior ETV therapy may be associated with a higher likelihood of NUC restart after discontinuation. During the two-year follow-up, only three patients lost HBsAg.

None of the patients experienced biochemical flares (ALT > 2 × ULN) or signs of hepatic decompensation during follow-up. Furthermore, none of the patients developed HCC, decompensation, flare, or mortality during the complete two-year follow-up period. 

## Discussion

The highlight of this study is its demonstration that APASL-guided NUC discontinuation in carefully selected non-cirrhotic CHB patients appears feasible and reasonably safe in a small, carefully selected cohort with close monitoring. Nearly half of the cohort maintained sustained off-therapy virological remission at two years, with no cases of biochemical flare or hepatic decompensation observed. Importantly, HBeAg-negative patients had significantly higher sustained remission rates than HBeAg-positive patients, consistent with existing literature.

Our findings align with the society guidelines’ emphasis on patient selection and post-cessation vigilance. AASLD 2018 discourages routine discontinuation in HBeAg-negative patients without robust follow-up capacity, whereas EASL (2017; updated 2025) supports a case-by-case approach with explicit retreatment thresholds and intensified early monitoring [8-10].

Emerging Indian data also speak of immune activity after NUC withdrawal and potential “rescue” strategies. Small Indian cohorts suggest that immune flares after cessation can be immunologically productive and, in selected HBeAg-negative patients who relapse, adjunctive peg-interferon may accelerate HBsAg decline or loss - hypotheses consistent with mechanistic studies of post-cessation immune restoration [14-16]. While our cohort did not include adjunctive interferon, incorporating biomarkers recommended by EASL/AASLD (e.g., end-of-treatment HBsAg and, where available, hepatitis B core-related antigen (HBcrAg)) may further enrich candidate selection and refine Indian care algorithms [8-10]. 

Hall et al. [1] and Seto et al. [2] reported high rates of early virological relapse after discontinuation, often exceeding 90% within the first year. However, sustained responders frequently had low baseline HBsAg levels. Our lower relapse rate (48.3% at three months) may be attributed to strict adherence to the APASL stopping criteria and intensive follow-up. The STOP-NUC trial by van Bömmel et al. [4] showed a 10% loss of HBsAg after cessation, compared with 0% in continuous therapy, emphasising the potential immune-restoration benefit of stopping treatment. While our study did not document HBsAg loss, this likely reflects the small sample size and lack of quantitative HBsAg monitoring.

The multinational RETRACT-B study [5] highlighted the predictive value of low end-of-treatment HBsAg levels (<100 IU/mL in Asians) for achieving HBsAg loss. The APASL guidance [6] includes a broader range of candidates than EASL/AASLD, which may help explain regional differences in relapse and remission rates. Our findings are consistent with the systematic review by Papatheodoridis et al. [3], indicating that sustained off-therapy remission can be achieved in 40%-50% of carefully selected patients, with severe adverse outcomes being rare in non-cirrhotic cohorts.

Additional studies support these observations. Hadziyannis et al. [17] demonstrated that extended consolidation therapy in HBeAg-negative patients significantly lowers the risk of relapse, while Chi et al. [18] reported that close, early post-cessation monitoring is vital to prevent severe outcomes. Tseng et al. [19] found that combining quantitative HBsAg and HBcrAg can enhance patient selection. Terrault et al. [8] noted that virological relapse patterns vary by HBV genotype, with genotype C associated with a higher relapse risk, which may help explain regional differences in outcomes.

From a clinical perspective, our results reinforce the safety of finite therapy when coupled with close monitoring and prompt retreatment upon relapse. The lack of flares or decompensation in our study supports the APASL recommendation for intensive, early surveillance, especially within the first three to six months. Better outcomes in HBeAg-negative patients indicate that they remain the best candidates for discontinuation. Future research should include quantitative HBsAg and other predictive biomarkers to further refine patient selection. Furthermore, no statistically significant difference in relapse was observed between patients treated with tenofovir and ETV (p = 0.09). However, there was a trend toward a lower relapse rate in the tenofovir group compared with the ETV group (5/15 vs. 9/14), warranting further evaluation in larger, multicentric studies.

The strengths of our study include strict eligibility criteria based on APASL guidelines, enhancing internal validity for evaluating finite therapy strategies in suitable non-cirrhotic candidates; inclusion of both HBeAg-positive and HBeAg-negative patients, allowing subgroup comparisons relevant to clinical decision-making. To the best of our knowledge, the current study is the first from India to assess the outcomes of treatment cessation in non-cirrhotic hepatitis B patients. These findings are particularly relevant in the Indian setting, where a lower relapse rate was observed in patients who discontinued NUC therapy under structured monitoring. The absence of significant adverse events and the successful re-establishment of virological suppression upon retreatment with the same NUC suggest that a finite therapy approach may help reduce the overall financial burden.

The limitations of our study include a small, single-centre sample (n = 29), which limits the precision of estimates and the power of multivariable analyses; a short follow-up duration; limited external generalisability; and the retrospective study design. Additionally, key biomarkers, such as quantitative HBsAg and HBcrAg at the end of treatment, were absent, preventing risk stratification as recommended by recent studies. The study also featured limited virological characterisation and no contemporaneous control group receiving NUCs, which hinders direct estimation of absolute benefit-risk ratios relative to indefinite therapy, as well as imaging and elastography after treatment stoppage.

## Conclusions

In this single-centre cohort of non-cirrhotic CHB patients meeting the APASL stopping criteria, controlled discontinuation of NUC was feasible under structured surveillance. Nearly half of the patients maintained sustained off-therapy virological remission over two years, while all those with early reactivation achieved complete re-suppression after prompt retreatment, without biochemical flares, hepatic decompensation, or mortality. HBeAg-negative status was associated with a higher probability of sustained remission.

These findings support APASL-aligned finite therapy as a practical option in carefully selected patients, provided that early monitoring and clear retreatment thresholds are in place. Future work should incorporate quantitative HBsAg and HBcrAg, HBV genotype, and standardised elastography to refine selection and define optimal monitoring intervals. However, larger, prospective, multi-centre trials are needed to validate these findings before widespread clinical implementation.
